# How Do Households Respond to Unreliable Water Supplies? A Systematic Review

**DOI:** 10.3390/ijerph13121222

**Published:** 2016-12-09

**Authors:** Batsirai Majuru, Marc Suhrcke, Paul R. Hunter

**Affiliations:** 1 Norwich Medical School, University of East Anglia, Norwich Research Park, Norfolk NR4 7TJ, UK; missbatsi@hotmail.com; 2 Centre for Health Economics, University of York, Heslington, York YO10 5DD, UK; marc.suhrcke@york.ac.uk; 3 Department of Environmental Health, Tshwane University of Technology, Private Bag X680, Pretoria 0001, South Africa

**Keywords:** developing countries, water supply, reliability, coping strategies

## Abstract

Although the Millennium Development Goal (MDG) target for drinking water was met, in many developing countries water supplies are unreliable. This paper reviews how households in developing countries cope with unreliable water supplies, including coping costs, the distribution of coping costs across socio-economic groups, and effectiveness of coping strategies in meeting household water needs. Structured searches were conducted in peer-reviewed and grey literature in electronic databases and search engines, and 28 studies were selected for review, out of 1643 potentially relevant references. Studies were included if they reported on strategies to cope with unreliable household water supplies and were based on empirical research in developing countries. Common coping strategies include drilling wells, storing water, and collecting water from alternative sources. The choice of coping strategies is influenced by income, level of education, land tenure and extent of unreliability. The findings of this review highlight that low-income households bear a disproportionate coping burden, as they often engage in coping strategies such as collecting water from alternative sources, which is labour and time-intensive, and yields smaller quantities of water. Such alternative sources may be of lower water quality, and pose health risks. In the absence of dramatic improvements in the reliability of water supplies, a point of critical avenue of enquiry should be what coping strategies are effective and can be readily adopted by low income households.

## 1. Introduction

2015 marked the end of the Millennium Development Goal (MDG) era, in which part of the development agenda was to halve the proportion of the world’s population without sustainable access to safe drinking-water. The target was met five years ahead of schedule, and by 2012, an estimated 89% of the world’s population had access to safe water [1]. However, the announcement of this achievement was met with caution, as it had long been recognised that the indicator used to monitor progress against the target—“use of an improved source”—did not adequately reflect the quality of service, including the safety and reliability of water supplies [2,3].

Systematically collected data on reliability of water supplies is limited, but it is estimated that at least 300 million people globally are served by water supplies that are available for less than 12 h a day—the vast majority of them in South Asia and sub-Saharan Africa [4]. At any given time, a third of hand-pumps across rural sub-Saharan Africa are thought to be non-functional [5]. In Mozambique for instance, it is thought that the national MDG target would have long been met, had the broken 22% of hand-pumps in the northern parts of the country been functional [6].

Concern about the impacts of unreliable water supplies has grown [7], and strengthened arguments for better metrics for the development agenda post-2015 [2]. While the topic remains relatively understudied, what is clear is that unreliability has adverse impacts on water quality [4,8], and health and welfare of households [7,9,10]. There is evidence to suggest that some strategies that households employ to cope with unreliable water supplies can in fact be detrimental. In Zimbabwe, urban households faced with chronic water supply interruptions resorted to digging shallow wells, whose contamination is now thought to have exacerbated the 2008–2009 cholera outbreak in the country [11,12].

Assuming that unreliable water supplies will continue to exist in many developing countries for the foreseeable future, there is a critical need to consider which, if any coping strategies will be most effective in ensuring that households are able to reduce risks to health, and obtain safe and sufficient quantities of water at an affordable cost. To date, much of the literature on coping with unreliable water supplies has focused on assessing costs of coping, and applying these coping costs as indirect estimates of willingness to pay for improved water supplies [13,14]. The usefulness of this approach may be limited if household strategies to cope with unreliable water supplies are to be understood beyond their implications on water demand and pricing options in service improvements. Consequently, this review seeks to address the following questions:

- How do households respond to unreliable water supplies?
- What factors influence the choice of coping strategies, and how are they distributed across socio-economic groups?
- What are the costs of coping strategies?
- What health and social outcomes are associated with coping with unreliability?
- How effective are these strategies e.g., are water quantity, quality and pressure needs met?

In attempting to answer these questions, we identify what is known and what is missing from the literature on coping with unreliable water supplies in developing countries. To our knowledge, this is the first review that has sought to synthesize the literature on this topic.

## 2. Methods

Before we turn to the methods used to address the review questions above, we begin with an overview of the conceptual background surrounding the notions of “reliability” and “coping” in the context of water supplies.

### 2.1. Conceptual Background on Water Supply Reliability and Coping

As pointed out in the 2012 update on the MDG water target, although there is wide recognition that reliability is an important aspect of water supply, there has been little consensus on how it should be defined, or consequently measured [1]. “Reliability” as considered in this review is a feature of water supply that is made up of several attributes. These attributes include: consistency with which water is supplied, e.g., 24 h a day, every day, or for part of the day on some days; the predictability of the supply, e.g., supply that is not continuous, but provided at regular intervals, or not continuous and at irregular intervals; and the pressure of the supply, e.g., pressure fluctuations may result in limited or no supply. This definition also extends to breakdowns in the supply systems itself, which we distinguish from intermittent/discontinuous supply, which may be more indicative of sub-optimal functionality than complete non-functionality. While the focus of this review is on reliability related to water supply infrastructure, it should be noted that environmental factors such as seasonality also impact on the reliability of water supplies.

Among the early attempts to systematically describe and analyse the impact of unreliability was a study funded by the World Bank to assess the extent of private costs that Nigerian firms incurred due to deficiencies in public services [15]. The methodology from this study was subsequently applied to household water supplies in India, Pakistan and Turkey around 1990–1992. From the three case studies, the authors suggested a conceptual framework and methods for analysing unreliability of water supplies and its impact on households [16], and summarised households’ responses to unreliability and the related costs [17]. This conceptual framework was later applied in another World Bank-funded study conducted in Azerbaijan in 1994 by Kudat et al. [18], and has shaped much of the often-cited work on the topic.

In their conceptual framework, Kudat et al. [18] and Humplick et al. [16] propose that as a commodity, water has three main attributes: quantity, quality and pressure. Where the water supply does not meet the optimum levels of these three attributes, the supply is said to be unreliable, and households will adopt strategies to mitigate risks from this unreliability.

The term “coping strategies” is used in a wide variety of contexts, and can have specific connotations in some disciplines. For instance in social science literature, Davies [19] uses the term to describe strategies employed in response to crises. In this paper, we adopt the term “coping strategies” to describe households’ responses to unreliable water supplies. Kudat et al. [18] categorise these coping strategies as extending on Hirschman’s theory on “exit, voice and loyalty” [20], which describes consumer responses to deteriorating quality of good or services. Faced with chronic unreliability of supplies, households may “exit” the system by adopting strategies such as drilling wells, installing large capacity storage tanks (thus mimicking continuous supply), or even relocating to areas where water supply is more reliable. The “voice” strategy includes complaints and protests to water utilities or local authorities. Households could also be “loyal”, and engage in accommodative strategies such as rescheduling activities to when water is actually available, and reducing quantity of water they use.

The authors also suggest that determinants of coping strategies can be grouped into three levels: (i) household level, e.g., socio-economic status, gender, age structure; (ii) settlement level, e.g., water service level, geographic location; and (iii) national level e.g., privatisation of water supply sector, regulatory environment [16,21]. Subsequent studies have drawn upon the work of Kudat et al., focusing mainly on measuring the costs of coping strategies [22,23,24] as indirect estimates of willingness to pay for more reliable services.

### 2.2. Literature Search Methods and Selection Criteria

Literature searches were conducted in CINAHL EBSCOHost, Embase Ovid, PubMed Central, Scopus, ScienceDirect, Scirus and Web of Knowledge using the following search terms: TITLE-ABSTR-KEY (“water supply” OR “safe water” OR “drinking water” OR “domestic water” OR “household water” OR “water point”) AND TITLE-ABSTR-KEY (reliab* OR sustainab* OR availab* OR function* OR regular OR access OR intermitten* OR interrupt* OR constant OR continu* OR consistent OR “operation and maintenance” OR breakdown) AND TITLE-ABSTR-KEY (cope OR coping OR “coping strategies” OR avert OR “averting behaviour” OR respond).

Searches were also conducted in the Google and Google Scholar search engines, where we checked the first 50 hits for potentially relevant papers. Papers obtained from the search were included for review if they met the following criteria:

- Report on coping strategies relating to unreliability in the performance or functionality of water supply systems
- Report on domestic/household water supply
- Report data from developing countries
- Based on empirical research

Although physical unavailability/scarcity of water contributes to unreliability of water supplies the world over, in many developing countries a significant proportion of the problem lies in poor water resource management, decaying infrastructure, and poor accountability [25]. Thus, our review focuses on responses to unreliability relating to the performance or functionality of water supply systems and distribution networks. Developing (i.e., low- and middle-income) countries were defined as per the World Bank classification of 2012. Studies that only listed coping strategies with no further supporting detail were excluded. Full texts of papers in English whose abstracts and titles met the criteria were retrieved and reviewed in detail for study quality and findings. The reference lists of relevant papers were also checked for other potentially relevant papers.

### 2.3. Quality Appraisal

The studies included in this review are from a variety of disciplines and therefore represent widely different methodological approaches. As such, the absence of uniform reporting presents a challenge to any rigorous appraisal of study quality. Our appraisal of study quality was based on an approach adapted from Hellebrandt et al. [26]. The approach is largely based on assessment criteria suggested in the Cochrane and EPPI frameworks, which they adapted to suit the heterogeneity in study outcomes and study design encountered in their own review. Essentially, their appraisal covers most of the domains of the Cochrane and EPPI frameworks, but does not combine the measures of validity in an overall score or weight them in any way. Instead, the study strengths and weaknesses by are reported by domain, and include criteria such as clarity of the research question, adequate description of conditions, etc. Details on this approach are available online from their review protocol [26]. Our appraisal was similarly broad, and considered the clarity of research objective, description of methods used, description of water supply conditions, reporting of results, researcher bias, and any other issues that may influence study quality. In addition, we assigned a score to each of these criteria, and combined the score to categorise overall risk of bias as high, moderate, or low.

### 2.4. Categorization of Coping Strategies

As households often employ multiple coping strategies, categorizing the range of coping strategies assessed is a useful approach. Subsequent to the early studies by Kudat et al., the four studies that have categorized the range of coping strategies assessed have done so on the basis of: actual actions taken by households, e.g., storing, pumping, collecting, purchasing or treating water [24,27], outcomes ensuing from the various actions, e.g., adaptation, exiting from the formal water service [24]; or costs related to the various strategies, e.g., collection time, financial water costs, capital costs, diarrhoea treatment costs, and water treatment costs [28]. For the purposes of this review, we broadly categorize the coping strategies identified from the literature by the assumed outcome intended by these household actions as follows: enhancing/supplementing quantity of water available; accommodating unreliable supplies; improving water quality; and enhancing water pressure. We also consider a fifth category: collective action and voice. Although there is some overlap among these categories, we consider each of these in turn.

## 3. Results

**Figure 1:**
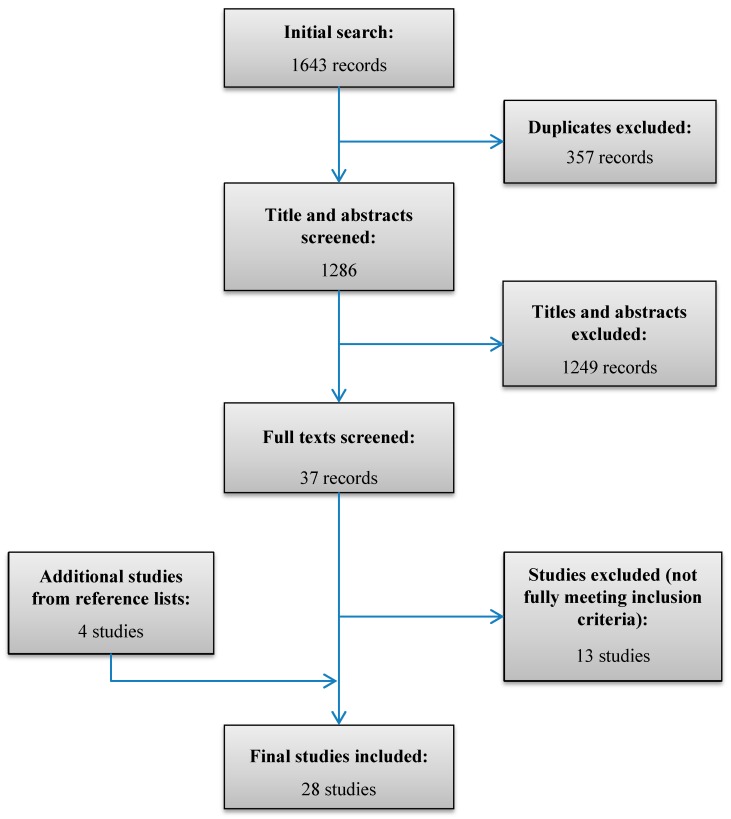
Flowchart of study selection process.

A total of 1643 papers were found from the database search. Of these, 357 were duplicates and the majority focused on agricultural or industrial water supply, or reported on coping with water scarcity due to drought, climate change, etc., and thus did not meet the inclusion criteria. Four studies were found from perusing reference lists of other studies, bringing the total number of studies reviewed to 28 (Figure 1).

The majority of studies (nine) were from South Asia, followed by Africa (eight) and the Americas and Caribbean (seven). The literature on coping strategies in rural areas is relatively thin; of the 28 studies reviewed, 22 were conducted in urban settings, two covered both rural and urban areas, three were conducted in rural settings (Table 1) and in one study the setting was not specified. All studies reported on cross-sectional data.

The literature on this topic is diverse; varying widely in terms of methodology and epistemology. Studies reviewed included qualitative and quantitative studies from disciplines such as engineering, urban planning, economics, anthropology and public health. A third of the studies estimated willingness to pay for improved/more reliable water services, and of these, six simultaneously measured coping costs and used them as indirect estimates of willingness to pay. Using the approach adapted from Hellebrandt et al. [26], 12 of the studies were categorised as having low risk of bias, 13 moderate risk and three high risk (Table 2).

Common sources of possible bias were in the methods used to address study objectives and the subsequent reporting of results not being clear. For instance, four of the studies [29,30,31,32] use convenience or snowball sampling techniques without any clear justification for doing so. Such samples are prone to selection bias as they are unlikely to be representative of the population [33].

### 3.1. Coping Strategies

The studies reviewed highlight that households often engage in multiple coping strategies; a total of 15 strategies were identified, and an average of five strategies reported per study (Table 3).

#### 3.1.1. Enhancing Water Quantity

Among the commonly reported strategies to enhance water quantity are: digging shallow wells or drilling boreholes; storing, and purchasing water.

Digging wells/drilling boreholes is reported in eleven studies. A set-up for drilled boreholes may include an electric pump connected to an overhead storage tank. Such high-capacity storage is reported in 21 studies, and capacities reported vary from 100 L [28], to over 1000 L [34]. Storage tanks can fill up automatically whenever the municipal supply becomes available [22]; by abstracting groundwater from boreholes [24]; or households may pay for tanker deliveries to fill up the tanks [22,30,35].

Households also harvest rainwater, and store it in such large capacity tanks, or in smaller vessels such as buckets and drums. Five studies [28,29,30,31,34] reported rainwater harvesting (Table 3), with storage capacities ranging from 50 to 200 L.

Water is also stored in smaller containers such as jerry cans, buckets and pots, as reported in 16 of the studies reviewed. However, the storage process for such smaller containers can be tedious. Households may be forced to stay up late, or wake up very early to fill storage containers while the supply is still available [30,35,36]. As noted in two studies [10,36], in cases where the supply does not follow a set schedule i.e., the supply is unpredictable, the feasibility of such storage is limited.

Purchasing of water is reported in half of the studies reviewed. Households purchase water from vendors or kiosks, neighbours who have private wells, or pay for container or tanker deliveries [22,27,30,35,37,38].

Illegal connections to the municipal water supply network in Dar es Salaam, Tanzania are reported in a study by Nganyanyuka et al. [30]. Households and water vendors had connected pumps and pipes to networks in areas with more reliable supply 2.5 km away, and sold the water that they had siphoned off these networks.

#### 3.1.2. Accommodating Water Supply Unreliability

Accommodative strategies identified include: collecting water from alternative sources, rescheduling domestic activities, reducing water use, or recycling water.

Collecting water from alternative sources is fairly common, and is reported in 17 studies. Alternative sources include communal taps or boreholes [24,27], springs, or surface water [30,36], trenches and burst water pipes [29]. Households may spend between 30 min [27], to three hours [28] collecting water from these alternative sources, depending on the distance to the alternative source, number of trips made and in some cases, time spent queuing [28,36].

A quarter of the studies reported households rescheduling domestic activities requiring a lot of water such as laundry, scrubbing floors etc., to days when water was available [14,24,30,31,36,39,40]. Strategies to reduce water use also include reducing intake of fresh fruit and vegetables that would need to be washed, reducing frequency of bathing and laundering [18] and flushing toilets only once a day [29]. In rural Botswana, Ngwenya and Kgathi [40] also found that the need to reduce water use posed significant challenges in caring for family members with HIV/AIDS-related illnesses. Households reduced the number of meals cooked per day, reserved potable water for drinking only by family members, reduced the number of baths given to the ill family members and kept soiled laundry until there was more water available. Households may also recycle water by reusing laundry or bath water for flushing toilets, mopping floors, watering vegetable gardens, etc. [18,24,30].

#### 3.1.3. Improving Water Quality

Unreliable water supplies often lead to poor water quality in various ways, including intrusion of contaminants into pipes when water pressure is low or supply interrupted, or when water collected from alternative sources is unsafe or re-contaminated during collection and storage [8]. Household water treatment through boiling, filtration or disinfection is reported in over half of the studies reviewed (Table 3). Boiling and filtration appear most common, with households using either one of the two methods or both [13,27,41].

Purchasing of bottled and/or sachet water is also reported in several studies [28,30,42,43]. Reported average weekly consumption of bottled water in three studies were: 3 L among households in rural Kenya [28]; 32 L in in urban Jordan [39] and 51 L in urban Mexico [42].

#### 3.1.4. Enhancing Water Pressure

We find some overlap in the needs that some coping strategies fulfil. For instance, the installation of overhead tanks enables households to store large quantities of water and also have such water flow into the water supply pipes at a reasonable pressure [22,24]. Other pressure-enhancing strategies include installing electric pumps to convey water from storage tanks, and installing motors directly onto municipal water connections to boost the water pressure [24].

#### 3.1.5. Collective Action and Voice

The strategies summarized above have focused on action undertaken within the household. Communities may also take collective action to address water needs, or employ the “voice” strategy; defined by Zérah [24] as “complaints, demonstration and associations”. However, the seven studies that mention these strategies provide little detail. In Dhaka, Bangladesh, Jamal and Rahman [44] found that in addition to adopting various coping strategies at household level, households also contributed towards the establishment of a communal tube well. In Turkey, households created community associations and pressured local authorities for better services [18].

### 3.2. Determinants of Coping Strategies Adopted

Socio-economic status and extent of unreliability of supply service are among the most significant determinants of coping strategies adopted. Households that are relatively wealthier, more educated and/or own the property they live on are more likely to engage in capital-intensive strategies such as drilling wells and/or installing storage tanks [18,22,24,28,45]. In Delhi, India, households were also more likely to adopt such strategies if the duration of water supply was very limited (defined in the study as less than four hours a day) [24]. Similarly, storage capacities were found to increase with income and less reliable supply [24,39,45]. With regards to level of service, households with piped connections were more likely to install storage tanks, while unconnected households relied on collecting water from alternative sources [22,24,27].

Three studies highlight that subjective perceptions of the reliability of water supplies also play a role. In cases where water supply unreliability is prolonged, households may actively anticipate poor reliability in their water supply, and construct houses that have in-built storage cisterns, or connect plumbing systems to drilled wells [18,21,34,45]. Similarly, perceived water quality influences water treatment behaviour, in addition to income and educational status [24,28,41]. In Kathmandu, Nepal, Katuwal and Bohara [41] found that wealthy households were more likely to use more than one treatment method compared to lower income households. The authors also noted some urban-rural differences in treatment behaviour, with the proportion of urban households that boiled water higher than those in rural areas.

Lower income households are more likely to accommodate unreliability and adopt time- and labour-intensive strategies such as rescheduling activities and collecting water from alternative sources [24,27,28]. In one study, collecting water from alternative sources comprised 56% of total coping costs for poor households, compared to 34% for wealthier households [27]. Compared to wealthy households, lower income households are also more likely to “voice” dissatisfaction with water supplies by demonstrating to local authorities [29].

### 3.3. Costs of Coping

While coping costs are reported in almost half of the studies reviewed, the actual components of the costs reported and assessment methods vary widely. From these, a total of nine cost components relating to both direct coping costs (e.g., purchasing water, installing storage tanks and digging wells) and indirect costs (e.g., opportunity costs of time spent collecting water, costs of water-related illness) were identified. However, the majority of studies reported on direct costs, and within these, costs related to purchasing water, or installing storage tanks. In rural Kenya, Cook et al. [28] found that the average cost of digging wells was US$400, while in Tanzania the cost varied between US$3000 and US$6000 [30]. Only four studies reported costs associated with household water treatment [13,23,27,28], and three studies reported costs associated with water-related illness [13,23,28].

Studies reporting coping costs also varied in the unit of analysis; costs were presented per unit purchased, per week, month, or year, and depending on the data distribution, as average or median costs. For instance, in Zimbabwe, water was purchased from vendors at a cost of 20-L per bucket, while in Guatemala, average monthly consumption of bottled was 51 L, at a cost of just over US$6 [43].

Three studies [24,27,28] rigorously analysed the distribution of coping costs across income groups, and found that direct costs related to drilling wells and purchasing and treating water comprise the largest share of coping costs for wealthy households. In contrast, for low income households the time value of water collection comprises the largest share of coping costs [27].

Although collecting water from alternative sources is widely reported, most studies only report on the time spent collecting water, and only three [22,27,28] estimate the value of this travel time. In Kathmandu, Nepal, time spent collecting water from alternative sources comprised 45% of coping costs, on average. For households without piped connections these time-related costs were even higher, at 65% [27]. In Kenya, the median time-related costs for households without piped connections were equivalent to 6% of monthly income [28].

In the few studies that reported ratios of coping costs to income, such costs were unevenly distributed across income groups. For instance, mean monthly coping costs in Kenya were US$38, while the median costs were almost half, at US$21 [28]. These median costs were equivalent to 12% of income, while in Nepal mean coping costs were equivalent to 1% of monthly income [27]. Although lower income households generally incur lower direct coping costs, these costs still comprise a higher proportion of their income. In India, Zérah [23] found an increase in the income/ratio for low income households, and coping costs comprised 15% of income for lower income households, compared to 1% for wealthy households.

Households with piped connections may still be charged with water utility bills, in addition to their coping costs. In one study [29] households went for more than a month without municipal water supply, but were still required to pay fixed water charges which were mandatory for households with piped connections. Several studies compared coping costs to utility water bills, and found that at a minimum, coping costs were equivalent to utility water bills [22], but could be as much as double [27], or six times the bill amounts [23]. In two studies [23,46] the annual aggregated coping costs incurred by households exceeded the water utility’s budgeted cost of supply.

How closely do coping costs reveal households’ willingness to pay (WTP) for improved services? Evidence from the eight studies that included assessments of WTP for improved/more reliable water supplies was rather mixed. In a WTP survey in Nepal, households were offered a more reliable (24-h) service by a private operator providing 500 L a day with fair metering. The monthly mean WTP for this hypothetical service was almost 6 times greater (US$17) than mean coping costs (US$3) [27]. While Pattanayak et al. [27] acknowledge that relying on coping costs alone would significantly underestimate households’ WTP, they also argue that these costs serve as useful lower bound estimates of WTP.

In contrast to the findings in Nepal, WTP was lower than coping costs in three studies in India and Pakistan [13,22,46]. While this may be due to affordability constraints, a study by Virjee and Gaskin [34] suggests that this low WTP may arise because of an erosion of household confidence in the water system, due to chronic unreliability. In a survey conducted in Trinidad in 1994, Mycoo [14] had found WTP estimates equivalent to coping costs, and twice the amount of the water bills at the time. However, a later study in 2003 by Virjee and Gaskin [34] in Trinidad and Tobago found that WTP had decreased, and was significantly lower than water bills. In the study, 68% of households had invested in large storage tanks, compared to 37% at the time of the study by Mycoo. The average capacity of these storage tanks (610 gallons) allowed households to simulate continuous water supply, resulting in indifference to proposed water service improvements [14].

### 3.4. Health and Social Outcomes Related to Coping

We find no studies aimed specifically at assessing health and/or social outcomes related to coping strategies that households employed. However, several studies suggest that some strategies compromise water safety, hygiene and nutritional practices, and emphasise gender inequalities.

A study by Caprara et al. [35] in Brazil highlighted that poor water storage practices facilitated breeding of the dengue vector *Aedes aegypti*. In Maputo, Mozambique, Matsinhe et al. [47] found that most storage tanks were poorly maintained, and households did not clean or disinfect tanks regularly. Storage tanks were oversized for households’ water demands, resulting in long storage times that supported microbial regrowth. In another study in Zimbabwe, households had dug shallow wells that were subsequently contaminated by sewerage pipe bursts and surface run-off [29].

Three studies noted that reducing water use as a coping strategy compromised hygiene and sanitation practices such as bathing, household cleaning, and flushing toilets [18,29], nutritional practices, and ability to care for family members suffering from HIV [40].

The studies reviewed also highlight that women and children bear the primary burden of collecting water from alternative sources [29,31,40], with one study reporting that women got up at 4 am to queue for water from boreholes in the area [29]. In another study, women complained about having “head pains” and chest pain from carrying water from alternative sources [31].

### 3.5. Effectiveness of Coping Strategies Adopted

Few studies assessed the effectiveness of coping strategies in meeting household water needs, with the exception of the study in Delhi, India, by Zérah [24], who assessed quantity of water stored in relation to coping strategy adopted. Stored water quantities were largest for households who had tanks linked to drilled wells (200 L per capita per day (Lcd)); followed by households with tanks linked to municipal supply (150 Lcd); and smallest for households collecting water from alternative sources (30 Lcd). Similarly, Cook et al. [28] noted that households who collected water from alternative sources used smaller quantities of water than those who had drilled wells, although no details are provided on the relative quantities. A few studies suggest that among those households collecting water from alternative sources, having means of transport (donkey carts, wheelbarrows, bicycles etc.) allows them to collect larger quantities and spend less time doing so [28,29,40].

Installing overhead storage tanks or having electric pumps attached to the tanks allows households to simulate continuous water supply, and has the added advantage of enhancing water pressure and general convenience [18,21,24,34,46].

It is unclear from the studies reviewed whether the “voice” strategy is effective in pressuring water utilities to improve services. While none of the studies sought to address this question, the available evidence suggests that such strategies are rarely employed in an organised manner at a large scale, and therefore households may not believe that anything would come out of it [24,29].

## 4. Discussion

Households faced with unreliable become responsible—to varying degrees—for the abstraction, treatment and “supply” of their own water, and adopt strategies such as drilling wells, storing or treating water, or collecting it from other sources.

Fundamentally, the adoption of any coping strategy carries costs; whether direct monetary expenses associated with e.g., drilling wells, or indirect costs of strategies such as collecting water from alternative sources. It is therefore unsurprising that household income is among the main determinants of coping strategies adopted; the link between socio-economic status and access to water and sanitation is known [48].

Wealthy households typically engage in capital-intensive strategies such as drilling boreholes and installing water storage tanks, and are also more likely to treat their water. These strategies appear to be not only long-term, but also comprehensive; providing (to some extent) quantity, quality and convenience. In contrast, low income households appear to focus on addressing the more immediate quantity problem [30], and engage in labour- and time-intensive strategies such as collecting water from alternative sources. These strategies not only cost more over the longer term due to the ongoing effort they require, but perversely yield smaller quantities of water.

Although none of the studies explicitly sought to assess health or social outcomes, the available literature suggests that some strategies compromise water quality, sanitation and hygiene practices and reinforce gender inequalities. Drilling wells can have important implications on the quality and depletion of groundwater [24,49]. Moreover, an increasing reliance on pit latrines may likely further compromise groundwater quality [50]. In areas where geo-genic contaminants such as arsenic and fluoride pose significant health risks, the drilling of wells as a coping strategy may actually be counter-productive. Installing motors directly onto municipal water connections to boost the water pressure increases risk of intrusion of contaminants [4], and lowers water pressure for other households connected to the network.

An important finding from this review is the significance of time as a major component of coping costs, particularly for low income households. Women and girls bear the primary burden of water collection, and the time spent has far reaching consequences relating to productivity losses, poor school attendance and educational attainment [3,51] and ultimately, sustained impoverishment. Having to collect water from alternative sources not only results in time losses, but may negate potential health benefits as well, depending on the source used [9].

Of interest is that collective action and “voice” are relatively uncommon. Collective action such as households contributing to the set-up of communal water sources would perhaps be indicative of a willingness to seek long-term solutions that provide economy of scale, particularly for households of lower socio-economic status. However, Manzungu et al. [52], warn that collective action may not be easily achieved where the state has previously assumed the role of service delivery, as in many of the studies reviewed herein. Further, collective action may require levels of social cohesion and trust that other households will pay in their contribution [53], as well as coordination, leadership and start-up funds [29,30] that should not be assumed to exist.

Studies of coping costs, WTP and current water bills highlight that coping costs are at a minimum, equivalent to bills from water utilities, and in some studies, WTP was higher than the coping costs that households were already incurring. This raises the question as to whether coping costs could be redirected to water utilities and facilitate system improvements. The answer may not be straightforward, for a couple of reasons. First, the studies suggest that chronic unreliability results in distrust of water utilities, and WTP decreases among wealthy households as they engage in long-term strategies that allow them to “exit” from the system. Such households would need convincing that the system will improve [24,34]. As Galaitsi et al. [54] note, the situation is akin to a vicious circle: supply is unreliable, consumers disengage, utility loses revenue, and the service further deteriorates. Second, for low income households whose main coping currency is time, it is not clear how readily they can convert time expenditures arising from water collection to monetary expenditures on water bills [28].

In many developing countries there is weak oversight of alternative supply options such as drilling wells [39], water vending [55] and sachet water [56]. In the few cases where there are regulations in place, there is an inordinate focus on pricing [57], or regulations are highly fragmented [58]. As households assume more responsibility for their water, they move further into this regulatory vacuum. The main challenge for water authorities therefore is how this vacuum can be filled in a manner that takes equal account of the need that alternative providers meet for consumers faced with unreliable supply, and protects consumers’ interests with regards to the safety and cost of the water provided.

In the most fundamental sense our review is limited by the current lack of a universally agreed upon definition of water supply reliability. Although we have attempted to capture the various terminology used in the literature on reliability in our search terms, the studies retrieved must be considered in the light of this limitation. In addition, there may have been studies assessing some coping strategies that do not state this in the abstract of the paper and would consequently be missing from the review.

As this review was limited to studies reported in English, there may be studies in other languages that may have been missed. Thirdly, the literature on coping with unreliability is widely dispersed across various disciplines. While this is not necessarily a limitation, it does bring up significant variations in the reporting structure and consequently makes it difficult to synthesise results in a systematic manner. In this review we used a thematic approach to analyse the literature. The heterogeneity in study methods and reporting of study outcomes also made appraisal of study quality through common appraisal tools less useful. Although we have tried to deal with this by adapting an existing appraisal approach, this only allows for broad indications of study quality.

The above limitations notwithstanding, the findings of this review have several implications for research and policy in the water sector. From a research perspective, four issues are worth noting that could be addressed in future studies. First, only five of the 28 studies were conducted in rural settings. A possible explanation for this geographic bias towards urban areas may lie in that a number of studies reviewed were mainly aimed at assessing demand for improved water services. The perception that willingness and/or ability to pay amongst rural households is low [59] may influence whether studies are conducted in these areas. Rural households still comprise the majority of the population in many developing countries [60], and additional studies in these settings would be useful for a nuanced understanding of households’ coping strategies.

Secondly, the literature reviewed is characterised by a reliance on mainly small-scale cross-sectional studies. Although such studies have been useful in describing the typology of coping strategies and their determinants, they provide little insight into the long-term implications of strategies adopted. There is therefore a need for rigorous, well-designed longitudinal studies in this area.

Although almost half of the studies reported on coping costs, there were considerable variations in study methods that limited the ability to summarize such figures in any meaningful way. For instance, among the few studies that reported disaggregated costs by income group, the ratios of coping cost to household income ranged from 1% [27], to as high as 12% [28]. Two possible explanations may be differences in: (i) the specific strategies that households engage in and the associated costs within their specific locale (e.g., drilling wells may be cheaper in an area where the water table is high); and (ii) the range of coping costs assessed and methodologies applied. These point to the need for future studies to consider the full complement of strategies that households employ, and employ a common methodology to assess coping costs, that would facilitate more meaningful comparisons across settings. As coping costs appear unevenly distributed across income groups, median figures may reflect such costs more accurately. Given the significance of time in coping costs, we would also suggest that future studies should rigorously assess this cost component.

Fourth, none of the studies reviewed explicitly sought to evaluate the implications of strategies on household health and welfare. Essentially, more research needs to ask the questions: are strategies effective, in terms of ensuring that households are able to reduce risks to health, and obtain safe and sufficient quantities of water at an affordable cost? If so, what conditions are necessary for the adoption of such strategies? Directly assessing these issues will allow for the development of a sound decision base for coping strategies that can be promoted to support households faced with unreliable water supplies.

Finally, unreliable water supplies do not only affect households, but also institutional settings, such as health care facilities, schools, etc. A recent global report highlighted that the estimated water coverage in health care facilities drops by almost half, when reliability and safety of supplies are considered [61]. Therefore, urgent attention should also be given to coping strategies that are being employed in these settings and how they can be optimized.

Developing feasible solutions is perhaps a more challenging task than diagnosing the nature and extent of the problem. Parallel to the need to improve evidence on which coping strategies are effective is the translation of such evidence to mitigatory action that can be undertaken at both the household level and water supplier level. At household level, effective mechanisms of disseminating information and capacitating households on water management practices are key. For instance, the findings of this review suggest that at a minimum, households may benefit from interventions aimed at improving water treatment and storage practices, including maintenance and cleaning of storage tanks. Evidence also suggests that households would benefit from predictable intermittency, in which water supply is not continuous, but follows a predictable schedule [54]. In particular, a study by Baisa et al. [10] noted welfare gains resulting from the supply of water at regular intervals. Thus, for water suppliers this may be a potential solution where continuous supply may not be immediately feasible in the short term. As poor households are often the ones least able to cope, such interventions could be targeted towards mitigating water supply shortages amongst these sections of the society.

At the level of water service providers, solutions to address unreliability are need. While restructuring tariffs can potentially raise funds for financing system improvements [48], households are unlikely to pay for services if they have disengaged from the system, or distrust that the system improvements will last. Thus, on the side of water service providers, improved governance would be critical, and lasting solutions are those that improve accountability in water management and service delivery at various spheres of government [25,62].

Post-2015, the Sustainable Development Goals (SDGs) succeed the MDGs, and have placed emphasis on corresponding with human rights criteria of quality, availability, accessibility, acceptability and affordability, as well as increasing the quality and resolution of data used for tracking progress towards the goals. In the context of SDG target 6.1, which aims to achieve universal and equitable access to safe and affordable drinking water for all by 2030 [63], indicators on water quality, reliability, collection time and affordability will need to be measured [64]. While some of these indicators cannot be monitored immediately post-2015, their monitoring in the medium to long term will mean that reliable global data will become available [64]. Assuming that there is some truth to the old adage that “what gets measured gets done”, this may present important opportunities to comprehensively tackle the problem of unreliable water supplies. Given the findings of this review on the coping costs (direct and indirect) that unreliable water supplies impose on households, we would add that the “affordability” criterion consider household expenditures on water that are related to unreliable water supplies.

## 5. Conclusions

Perhaps the most obvious finding emerging from this review is that unreliable water supplies impose significant coping burdens on households. In particular, the poorest sections of society suffer most from the impacts of unreliable water supplies and rely on coping strategies that are labour and time-intensive. Consequently the poorest sections of society may be missing out on the health and other benefits of access to safe water supplies even when they are reported as being served by improved supplies. As such, efforts aimed at mitigating unreliability of water supplies should target them.

There is considerable heterogeneity in study methods and disciplines from which studies on coping emanate, limiting the ability to draw quantitative patterns. The findings of this review thus point to a significant gap in the evidence base on which strategies are effective, how sustainable they are, and the conditions under which they can be adopted. There is therefore a need to step up research into large scale, rigorous evaluations in this area.

Taken together, the findings of this review support a key shift from focusing on *coverage* of water supplies to improvement of the *quality of service*, and in particular, water supply reliability. Such a shift is imperative to the attainment of SDG target 6.1, if indeed the goal is to ensure universal and equitable access to safe and affordable drinking water for all.

## Figures and Tables

**Table 1:** Assessment of bias.

Author(s), Year	Clarity of Research Objective	Description of Study Methods	Description of Conditions	Reporting of Results	Researcher Bias	Other Validity Issues	Score	Overall Risk of Bias
Baisa et al., 2010 [10]	2	2	2	2	1	1	10	Low
Dutta et al., 2005 [13]	2	2	2	1	2	1	10	Low
Mycoo, 1996 [14]	2	2	2	1	2	1	10	Low
Humplick et al., 1993 [16]; Madanat & Humplick, 1993 [17] *	2	1	1	1	1	0	6	High
Kudat et al., 1993 [18]	2	1	2	1	1	0	7	Moderate
Kudat et al., 1997 [21]	2	1	2	1	1	0	7	Moderate
Choe et al., 1996 [22]	2	2	2	1	2	1	10	Low
Zérah, 1998 [24], 2000 [24] *	2	1	2	2	1	1	9	Moderate
Pattanayak et al., 2005 [27]	2	2	2	2	2	1	11	Low
Cook et al., 2016 [28]	2	2	2	2	2	1	11	Low
Chaminuka & Nyatsanza, 2013 [29]	2	1	1	1	0	0	5	High
Nganyanyuka et al., 2014 [30]	2	1	1	1	1	1	7	Moderate
Olsson & Karlsson, 2010 [31]	1	1	2	2	1	0	7	Moderate
Potter & Darmane, 2010 [32]	2	1	2	2	1	0	8	Moderate
Virjee & Gaskin, 2010 [34]	2	2	2	2	2	1	11	Low
Caprara et al., 2009 [35]	2	2	2	2	2	1	11	Low
Smiley, 2016 [36]	0	2	2	1	1	1	7	Moderate
Gulyani et al., 2005 [37]	2	1	2	1	2	1	9	Moderate
Widiyati, 2011 [38]	2	1	2	1	1	1	8	Moderate
Gerlach & Franceys, 2009 [39]	2	2	2	1	2	0	9	Moderate
Ngwenya & Kgathi, 2006 [40]	2	2	2	2	0	0	8	Moderate
Katuwal & Bohara, 2011 [41]	2	2	2	1	1	1	9	Moderate
Vásquez et al., 2009 [42]	2	2	2	2	2	1	11	Low
Vasquez & Espaillat, 2016 [43]	2	2	2	2	2	1	11	Low
Jamal & Rahman, 2012 [44]	1	0	1	1	1	0	4	High
Vásquez, 2012 [45]	2	2	2	2	2	1	11	Low
Altaf, 1994 [46]	2	1	1	2	2	1	9	Moderate
Matsinhe et al., 2014 [47]	2	1	2	1	1	1	8	Moderate

Low risk of bias 10–12; Moderate risk of bias 7–9; High risk of bias: ≤6. * these studies were based on the same dataset and were considered as one study during the review.

**Table 2:** Description of studies included in the review.

Author(s), Year	Study Objective(s)	Setting, Location	Type of Supply	Sample
Baisa et al., 2010 [10]	Estimate the welfare costs of unreliable water supply	Urban, Mexico	Piped connections	Mexican National Household Survey of Income and Expenditure; sample size not reported
Dutta et al., 2005 [13]	Examine how much money people in unplanned areas are willing to pay to support a policy that provides them with a better and reliable water supply	Urban, India	Piped connections	Multistage stratified random sampling of 1100 households
Mycoo, 1996 [14]	Examine cost recovery potential based on household willingness to pay more for an improved service and water pricing	Urban, Trinidad	Piped connections	Stratified sample of 420 households, stratified according to slope, land elevation and income. Survey of households and interviews of professionals in the water sector
Humplick et al., 1993 [16]; Madanat & Humplick, 1993 [17]	Present a model and methods for analysis of households’ responses to unreliable water supply	Urban, Pakistan and Turkey	Piped connections and communal standpipes	Case studies with sample of 30 households in Turkey and 900 in Pakistan
Kudat et al., 1993 [18]	Assess households’ responses to unreliable water supply	Urban India, Pakistan and Turkey	Piped connections and communal standpipes	Case studies of 30 households in Turkey, 900 in Pakistan and 1011 in India
Kudat et al., 1997 [21]	Present a methodology for a Social assessment for the World Bank’s Greater Baku Water Supply Rehabilitation Project	Urban, Azerbaijan	Piped connections	Rapid user surveys with 150 respondents and 400 respondents, consultations, stakeholder workshop
Choe et al., 1996 [22]	Estimate the real costs of an intermittent supply and predict how much people would pay for a continuous full-service metered supply	Urban, India	Piped connections and communal standpipes	Random-stratified cluster sample of 1100 households drawn from the 1995 electoral roll
Zérah, 1998 [23]; 2000 [24]	*Article 1(1998):* Measure the costs of unreliability; *Article 2 (2000):* Estimate the household demand for a service by assessing the actual behaviour adopted by households when they have to cope with an inadequate service	Urban India	Piped connections	Two stratified sample of 678 households in four zones of urban Delhi
Pattanayak et al., 2005 [27]	Evaluate how coping costs and willingness to pay vary across types of water users and income	Urban, Nepal	Piped connections, communal standpipes	Clustered sampling (probability-to-size), 1500 households in five municipalities of Kathmandu Valley
Chaminuka & Nyatsanza, 2013 [29]	Assess the causes and extent of water shortages and coping mechanisms used by affected residents in Harare	Urban, Zimbabwe	Piped connections	Convenience sample of 40 households obtained through snowballing techniques
Nganyanyuka et al., 2014 [30]	Document details of citizens’ strategies for accessing water in Dar es Salaam, Tanzania	Urban, Tanzania	Piped connections, communal standpipes	Purposive selection of two municipalities, and within these, four streets were selected, and 22 persons interviewed. Interviews were also conducted with municipal water engineers, “street leaders”, NGO staff and water vendors, who were identified through snowballing techniques
Olsson & Karlsson, 2010 [31]	Investigate how poor women cope with water problems and constraints to women accessing water	Rural, Zanzibar	Piped supply; yard taps; communal standpipe	Snowball sampling, with key informant, individual and group interviews with 19 participants
Potter & Darmame, 2010 [32]	Examine potential social equity dimensions in the use of water within Greater Amman	Urban, Jordan	Piped connections	Snowball sample of 25 low income and 25 high income households
Virjee & Gaskin, 2010 [34]	Ascertain the willingness to pay for changes in the level of service experienced by users	Urban and rural, Trinidad and Tobago	Piped connections and communal standpipes	The Central Statistical Office’s Continuous Sample Survey of Population sampling method was used to randomly select 1419 households, using a two-stage stratification scheme based on geography and labour force characteristics
Caprara et al., 2009 [35]	Investigate the relationship between socio-economic characteristics and community practices affecting *Aedes aegypti* vector ecology	Urban, Brazil	Piped connections and communal standpipes	Purposive sample of 204 households, with mixed methods descriptive case study approach
Smiley, 2016 [36]	Objective not specified; mention of highlighting variability of water access restrictions	Urban, Tanzania	Piped connections	3 wards selected out of a total of 90; surveyed conducted among 150 households, and interviews officials at ministries, water utilities and ward leaders
Gulyani et al., 2005 [37]	Examine current water use and unit costs and test the willingness of the unconnected to pay for piped water, yard connections, or an improved water kiosk (standpipe) service	Urban, Kenya	Piped connections, yard taps, communal standpipes	674 randomly selected households were interviewed in 22 sites in the three urban areas
Widiyati, 2011 [38]	Assess willingness to pay to avoid the cost of intermittent water supply in Bandung Municipality, Indonesia	Urban, Indonesia	Piped connections	Purposive selection of 31 sub-districts in Bandung, from which 200 households were surveyed
Gerlach & Franceys, 2009 [39]	Investigate the status of water supply service and regulatory arrangements with respect to poor and vulnerable consumers	Urban Jordan	Piped connections	Semi-structured interviews with key stakeholders, survey of 10 households, each in 9 selected poor neighbourhoods and small-scale surveying of private water tanker operations
Ngwenya & Kgathi, 2006 [40]	Investigate access to potable water in HIV/AIDS related home-based care households in five rural communities	Rural, Botswana	Piped connections, piped yard taps, communal standpipes	Two- stage stratified random sampling involving 39 caregivers, using structured and informal interviews, participant observation
Katuwal & Bohara, 2011 [41]	Estimate the effect of wealth, education, information, gender, caste/ethnicity and opinion about water quality on drinking water treatment behaviours	Rural and urban, Nepal	Piped connections and communal standpipes	Multi-stage sample of 2000 households, as part of the ‘Water Survey of Kathmandu-2005‘
Vásquez et al., 2009 [42]	Elicit household willingness to pay responses for safe and reliable drinking water in Parral	Urban, Mexico	Piped connections	Stratified random sample of 398 households in 6 geographic zones
Vásquez & Espaillat, 2016 [43]	Investigate households’ willingness to pay for improved water services	Setting not specified (San Lorenzo), Guatemala	Piped connections	Random sample of 500 households
Jamal & Raman., 2012 [44]	Explore impacts of gas and water supply crises and document coping strategies	Urban, Bangladesh	Type of supply not clear	Participatory rural appraisal applied to urban setting, sample size not reported
Vásquez, 2012 [45]	Investigate the relationship between perceptions of water supply reliability and household expenditures on water storage devices in León, Nicaragua	Urban, Nicaragua	Piped connections	Stratified random sample of 891 households in 8 geographic zones
Altaf, 1994 [46]	Describe household response to inadequate public piped water supply systems and highlight the economic implications of their efforts to improve level of service and reliability	Rural and urban Pakistan	Communal standpipes	Stratified random samples of 968 urban and 756 rural households
Matsinhe et al., 2014 [47]	Evaluate the effect of intermittency and household storage on the quality of drinking water distributed in Maputo	Urban, Mozambique	Piped connections	Water samples collected from water works, distribution centres, household tanks and taps in the network. Number of households sampled not reported

**Table 3:** Coping strategies identified from the literature.

	Baisa (2010) [10]	Dutta (2005) [13]	Mycoo (1996) [14]	Humplick (1993) [16]; Madanat (1993) [17]	Kudat (1993) [18]	Kudat (1997) [21]	Choe (1996) [21]	Zerah (1998; 2000) [23,24]	Pattanayak (2005) [27]	Cook (2016) [28]	Chaminuka (2013) [29]	Nganyanyuka (2014) [30]	Olsson (2010) [31]	Potter (2010) [32]	Virjee (2010) [34]	Caprara (2009) [35]	Smiey (2016) [36]	Gulyani (2005) [37]	Widiyati ( 2011) [38]	Gerlach (2009) [39]	Ngwenya (2006) [40]	Katuwal (2011) [41]	Vasquez (2009) [42]	Vasquez (2016) [43]	Jamal (2012) [44]	Vasquez (2012) [45]	Altaf (1994) [46]	Matsinhe (2014) [47]
Install storage tanks	✔	✔	✔	✔	✔	✔		✔	✔	✔		✔	✔	✔	✔			✔	✔	✔			✔	✔		✔	✔	✔
Store water in buckets, bottles etc.		✔		✔	✔	✔		✔		✔	✔	✔	✔	✔		✔	✔		✔	✔		✔		✔				
Collect water from alternative sources		✔		✔	✔	✔	✔	✔	✔	✔	✔	✔	✔				✔	✔		✔	✔	✔			✔			
Drill wells, install hand pumps				✔	✔	✔		✔	✔	✔	✔	✔							✔						✔		✔	
Purchase water		✔	✔	✔	✔	✔			✔	✔		✔		✔		✔	✔		✔	✔	✔		✔	✔	✔			
Install electric pump		✔		✔	✔		✔	✔		✔		✔		✔					✔	✔							✔	
Treat water (boil/filter/chlorinate)		✔	✔	✔	✔		✔	✔	✔	✔		✔	✔	✔	✔							✔	✔	✔	✔		✔	
Recycle water				✔	✔			✔				✔																
Use water sparingly				✔	✔							✔	✔				✔			✔								
Harvest rainwater										✔	✔	✔	✔		✔													
Reschedule activities			✔					✔				✔	✔				✔			✔	✔							
Protest/complain			✔	✔	✔	✔		✔			✔														✔			
Move to another house/area				✔	✔			✔																				
Install extra storage space				✔	✔																							
Set up illegal connections												✔																
Reduce baths and/or alter diet				✔	✔							✔									✔							
